# Expression of microRNAs in cerebrospinal fluid of dogs with central nervous system disease

**DOI:** 10.1186/s13028-018-0434-0

**Published:** 2018-12-18

**Authors:** Katia Marioni-Henry, Debiao Zaho, Pablo Amengual-Batle, Nina Marie Rzechorzek, Michael Clinton

**Affiliations:** 1 Royal (Dick) School of Veterinary Studies and Roslin Institute, University of Edinburgh, Easter Bush Campus, Roslin, Midlothian, EH25 9RG UK; 2 School of Veterinary Medicine, College of Veterinary, Medical and Life Sciences, University of Glasgow, Bearsden Road, Glasgow, G61 1QH UK; 3 Centre for Clinical Brain Sciences, University of Edinburgh, Chancellor’s Building, 49 Little France Crescent, Edinburgh, EH16 4SB UK

**Keywords:** Canine, Cerebrospinal fluid, microRNA, Neoplasms

## Abstract

In this pilot study we investigated the expression of 14 microRNAs in the cerebrospinal fluid (CSF) of dogs with neoplastic, inflammatory and degenerative disorders affecting the central nervous system (CNS). CSF microRNA (miRNA) expression profiles were compared to those from dogs with neurological signs but no evidence of structural or inflammatory CNS disease. Seven miRNAs were easily detected in all samples: miR-10b-5p, miR-19b, miR-21-5p, miR-30b-5p, miR-103a-3p, miR-124, and miR-128-3p. Expression of miR-10b-5p was significantly higher in the neoplastic group compared to other groups. There was no relation between miRNA expression and either CSF nucleated cell count or CSF protein content. Higher expression of miR-10b-5p in the neoplastic group is consistent with previous reports in human medicine where aberrant expression of miR-10b is associated with various neoplastic diseases of the CNS.

## Findings

MicroRNAs (miRNAs) are a class of small, non-coding RNAs of approximately 20–25 nucleotides in length that regulate gene expression at the post-transcriptional level [1]. MicroRNA molecules are extremely stable and miRNAs expressed in neurons can be found in cerebrospinal fluid (CSF) and serum [2]. For central nervous system (CNS) disorders, proximity to the diseased tissue and low cellular content make CSF the preferred fluid for analyses [1–3].

In human medicine, miRNAs in CSF have been considered as potential biomarkers for the early diagnosis and prognosis of various CNS neoplastic, inflammatory and degenerative diseases (e.g. gliomas, glioblastomas, metastatic brain tumours, multiple sclerosis, amyotrophic lateral sclerosis, Alzheimer’s disease and Parkinson’s disease) [1, 2, 4]. Recently, expression of miR-21 and miR-181c in the CSF of dogs with meningoencephalitis of unknown origin (MUO) has been reported [5].

The primary aim of this pilot study was to assess the expression of 14 miRNAs in CSF samples of dogs that underwent a diagnostic work-up for investigation of neurological signs. The secondary aim was to compare the miRNA profiles of dogs diagnosed with structural CNS disorders (i.e. inflammatory, neoplastic, and degenerative conditions affecting brain, spinal cord and meninges; see groups A, B and D in Table 1, which were combined for this comparison) to those of dogs without evidence of structural CNS disease (combined groups C and E); this second group included dogs with epilepsy but no evidence of a specific underlying cause (i.e. idiopathic epilepsy group C in Table 1) and dogs with neurological signs localised to peripheral nerves only (group E). A tertiary aim was to compare the expression of the miRNAs in CSF samples of dogs in each of the five groups listed in Table 1. The selection of the miRNAs was based on similar studies performed in human medicine that identified dysregulation of these 14 miRNAs in a variety of CNS neoplastic, inflammatory and degenerative diseases. Teplyuk et al. [6] reported increased levels of miR-10b and miR-21 in the CSF of patients with glioblastoma and brain metastasis of breast and lung cancer, compared with tumors in remission and a variety of non-neoplastic conditions; miR-21 and miR-19b were among the most highly upregulated miRNA in primary CNS lymphoma; miR-181c and miR-633 miR-128-3p miR-155-5p were upregulated in multiple sclerosis, dysregulation of miR-210 and MiR-922 miR-103a-3p miR-194-5p was reported in degenerative conditions [7] and dysregulation of miR146, miR155 and miR124 in epilepsy [8, 9].

**Table 1:** Clinical data for inflammatory condition group (A), neoplastic condition group (B), idiopathic epilepsy group (C), degenerative condition group (D), and other neurological disorders not affecting the CNS group (E)

Case	Signalment			Final diagnosis
	Breed	Age (years)	Sex	
A	Bernese Mountain Dog	1	FN	Steroid-responsive meningitis-arteritis
A	Medium size mixed breed	9	MN	Idiopathic polyradiculoneuritis
A	Bichon Frise	2	FN	Meningoencephalitis of unknown origin
A	Dalmatian	0.5	FE	Steroid-responsive meningitis-arteritis
B	American Bulldog cross	9	ME	Mixed glioma—brain^a^
B	Miniature Schnauzer	7	ME	Suspected histiocytic sarcoma—brain^a^
B	Flat Coat Retriever	8	MN	Histiocytic sarcoma—lumbar spinal cord^a^
B	Boxer	6	FN	Glioma—brain
C	Labrador Retriever	2	FN	Idiopathic epilepsy
C	Collie cross	8	FN	Idiopathic epilepsy
C	Collie	3	MN	Idiopathic epilepsy
C	Staffordshire Bull Terrier cross	7	FN	Idiopathic epilepsy
C	Giant Schnauzer	7	FN	Idiopathic epilepsy
C	Bearded Collie	6	FN	Idiopathic epilepsy
D	German Shepherd Dog	4	MN	Canine degenerative myelopathy—SOD1 homozygous
D	German Shepherd Dog	10	FN	Canine degenerative myelopathy—SOD1 homozygous^a^
E	Cavalier King Charles Spaniel	6	FN	Vestibular signs associated with otitis media-interna^b^
E	Border Collie	14	FN	Geriatric idiopathic vestibular disease^b^
E	Labrador Retriever	12	FE	Geriatric idiopathic vestibular disease^b^
E	Border Terrier	9	MN	Iliopsoas sarcoma^a^

SOD1: Canine degenerative myelopathy is a progressive neurodegenerative disease associated with the c.118G > A substitution in exon 2 of the canine superoxide dismutase 1 (SOD1) gene in German Shepherd Dogs and other affected canine breeds

*FE* female entire, *FN* female neutered, *ME* male entire, *MN* male neutered

^a^ Diagnosis supported by histopathology

^b^ Diagnosis supported by clinical examination, MRI findings, CSF analysis and cytology

The study was conducted in compliance with the guidelines of the Veterinary Ethical Review Committee of the Royal (Dick) School of Veterinary Studies of the University of Edinburgh (Approval Number 124.17; 20 November 2017). All animals were examined by a board-certified neurologist, and diagnoses were based on a combination of two or more of the following: clinical findings, magnetic resonance imaging (MRI), CSF analysis, and post-mortem examination (Table 1).

CSF collected via cerebellomedullary cisternal or lumbar puncture was centrifuged and the supernatant was frozen within 60 min of collection and stored at − 80 **°**C until further use. All samples were visually inspected and pink, red, or xanthochromic samples were excluded. CSF analysis and cytology was available for 17/20 samples (Table 2).

**Table 2:** Cerebrospinal fluid analysis (normal laboratory reference range for nucleated cells ≤ 5 cells/μL and for protein concentration ≤ 0.25 mg/dL for cisternal samples and ≤ 0.45 mg/dL for lumbar samples)

Groups	Available data	Nucleated cell count per μL (mean)	RBCs	Protein mg/dL (mean)
Inflammatory (group A)	4/4	1.1	2/4 rare	11.25
Neoplastic (group B)	2/4	4.4	Rare and moderate numbers	122
Idiopathic epilepsy (group C)	6/6	1.1	None	18.8
Canine degenerative myelopathy (group D)	2/2	2	None	20
Neurological signs not localised to CNS or meninges (group E)	3/4	0	None	30.6

RNA was isolated from a minimum of 250 µL and a maximum of 500 µL of CSF. RNA extraction and cDNA synthesis were performed using miRCURY LNA Universal RT microRNA PCR Starter Kit (Exiqon). Synthetic exogenous spike-in UniSp6 RNA was added into each sample. Real-time quantitative PCR (qRT-PCR) analysis was performed using the ExiLENT SYBR Green master mix (Exiqon) and the Stratagene Mx3000P real-time quantitative PCR system (Life Technologies). Relative expression was calculated as fold-change by the ΔΔCt method with UniSp6 as a reference followed by normalisation to the expression for sample C4 (control with idiopathic epilepsy) and to the volume of CSF available.

Cycle threshold (Ct) values are inverse to the quantity of target nucleic acid in the sample and high Ct values (above 35 cycles) can also be generated by degradation of the probe-based fluorophore by nonspecific amplification of background nucleic acids [10]. Accordingly, only C_t_ values < 32 were considered reliable and only miRNAs with C_t_ < 32 in the majority of samples were included in subsequent analyses. Analysis of variance (ANOVA) was used to determine if the expression of each miRNA differed among dogs with and without CNS structural disease and then among the five categories of disease (Table 1). A general linear model (GLM) was used to assess the relationship between CSF nucleated cell count and miRNA expression, and CSF protein content and miRNA expression. All statistical analyses were conducted with R version 3.2.2 [(2015–08–14) 2015, The R Foundation for Statistical Computing].

Seven miRNAs were amplified in all samples: miR-10b-5p, miR-19b, miR-21-5p, miR-103a-3p, miR-124, and miR-128-3p. The remaining seven miRNAs were not detected (C_t_ ≥ 32 in ≥ 50% of the samples): miR-146, miR-155-5p, miR-181c, miR-194-5p, miR-210, miR-633, and miR-922.

**Fig. 1:**
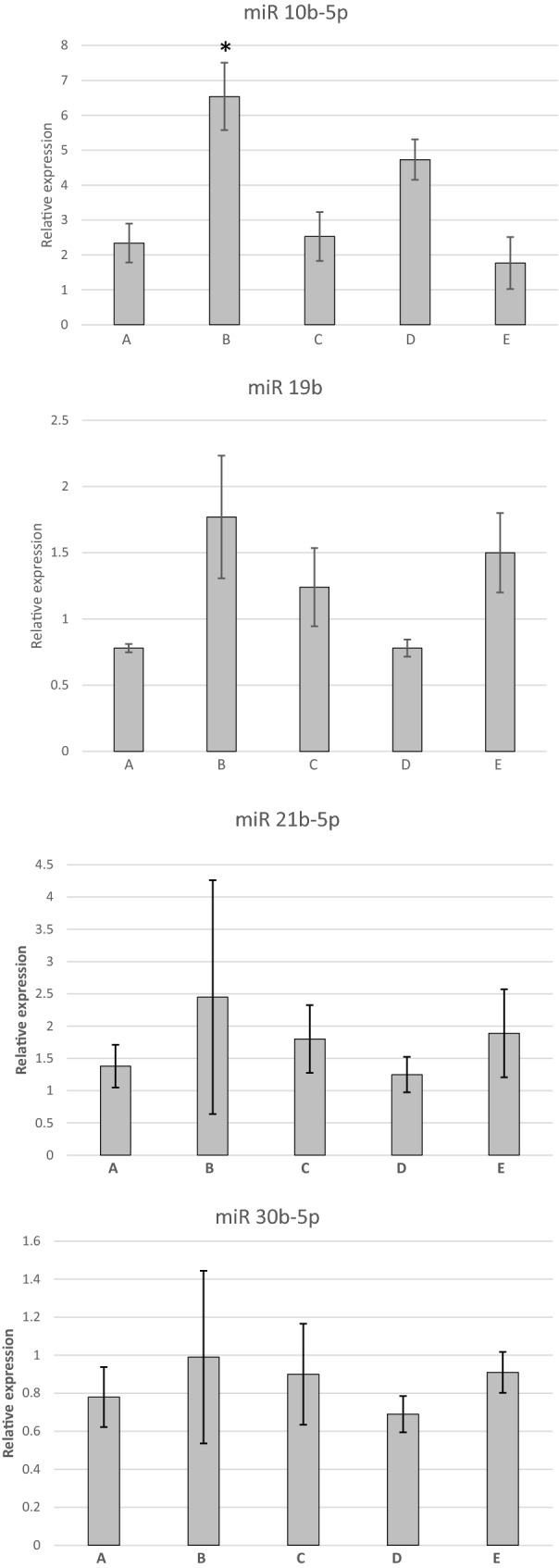
Relative expression of microRNA-10b-5p (miR-10b-5p), microRNA-19b (miR-19b), microRNA-21b-5p (miR-21b-5p), microRNA-30b-5p (miR-30b-5p) in cerebrospinal fluid (CSF) of dogs with inflammatory conditions (group A), neoplastic conditions (group B), idiopathic epilepsy (group C), degenerative conditions (group D), and other neurological disorders not affecting the CNS (group E). Error bars represent the standard error. *Statistically significant difference between groups (*P = 0.008)

**Fig. 2:**
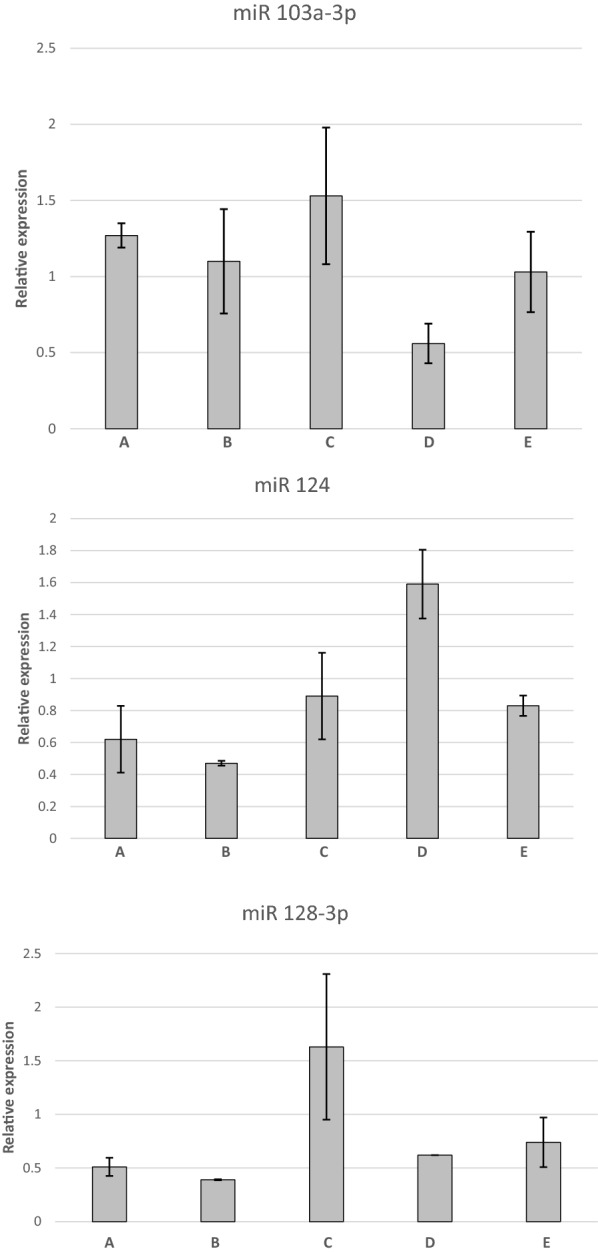
Relative expression of microRNA-103a-3p (miR-103a-3p), microRNA-124 (miR-124), microRNA-128-3p (miR-128-3p) in cerebrospinal fluid (CSF) of dogs with inflammatory conditions (group A), neoplastic conditions (group B), idiopathic epilepsy (group C), degenerative conditions (group D), and other neurological disorders not affecting the CNS (group E). Error bars represent the standard error. *Statistically significant difference between groups (*P = 0.008)

Expression of miR-10b-5p was significantly (P= 0.028), higher (mean 4.51 ± 0.79 SD, n = 10) in the group of dogs with CNS disease (combined groups A, B and D) compared to dogs without evidence of CNS structural disease (combined groups C and E; mean 2.28 ± 0.52 SD, n = 10). Expression of miR-10b-5p was also significantly (P= 0.008) higher in the neoplastic group (B) when compared to all other groups (Figs. 1, 2). No other significant differences in miRNA expression between groups were detected. However, the expression of miR-128 approached significance with a probability level of P = 0.074 in the idiopathic epilepsy group (C) when compared to all other groups (Figs. 1, 2).

No significant correlation (P > 0.05, GLM) was detected between CSF nucleated cell count and miRNA expression, or CSF protein content and miRNA expression.

Higher expression of miR-10b-5p in the neoplastic group is consistent with previous reports in human medicine; levels of both miR-10b and miR-21 were significantly increased in the CSF of patients with glioblastoma and brain metastasis of breast and lung cancer compared with tumours in remission and a variety of non-neoplastic conditions, while miR-10b was not detected in brain or CSF of non-cancer human patients [6].

A recent study investigating the expression of miR-21 and miR-181c in the CSF of dogs with MUO, found that the expression of miRNAs in the CSF, particularly miR-21, was correlated with the CSF nucleated cell count [5]. This finding was not replicated in our study using a GLM, possibly due to the low average nucleated cell count and the small sample size of this pilot study. In our study, the CSF samples were visually inspected prior to analysis and those presenting a reddish or xanthochromic discoloration were excluded to avoid contamination from peripheral blood. Therefore, acute forms of steroid responsive meningitis-arteritis (SRMA) associated with a high nucleated cell count and xanthochromic CSF would have been excluded by this study (Table 2). However, whilst highly inflammatory CSF samples do not pose a diagnostic challenge for SRMA, chronic SRMA cases can present with normal or only slightly elevated CSF protein content and mild pleocytosis [11, 12]. For more than 20 years researchers have been looking for biomarkers to improve the diagnostic and prognostic accuracy of SRMA [11, 12]. Based on our results, we believe that further studies investigating the use of upregulated miRNAs as biomarkers for chronic SRMA are warranted. For similar reasons, we decided to include cases of acute idiopathic polyradiculoneuritis (AIP) in our study. In the first instance, canine AIP is an inflammatory condition of the ventral nerve roots and peripheral nerves analogous to Guillain–Barré syndrome in humans where presence of CNS inflammation has been demonstrated by immunohistochemistry [13, 14]. Secondly, like chronic SRMA, canine AIP is diagnostically challenging since it presents with abnormal CSF analysis characterized by nonspecific elevated protein content and normal nucleated cell count [13]. Our primary objective was therefore to investigate the possibility of detecting upregulated miRNAs in the CSF of AIP cases as potentially useful diagnostic biomarkers.

The major limitations of this pilot study were the low sample size and the lack of a true control group of dogs without neurological signs; this to comply with UK legislation that requires that any medical procedure involving client-owned dogs must be performed for the direct benefit of the patient. Studies performed on human patients face similar limitations in regard to control groups, however, CSF is still considered the ideal source of miRNA to investigate brain disorders in human patients, due to a profile identical to that of the brain tissue and a less invasive and more accessible procedure compared to a brain biopsy [7].

## Conclusions

The expression of 14 miRNAs in the CSF of dogs with neurological disorders with and without structural CNS abnormalities showed that 7 miRNAs were consistently expressed in all samples. We also found a significantly increased expression of miR-10b-5p in the neoplastic group compared to other groups.

If our findings are replicated in a larger cohort, CSF miRNA expression analysis may prove to be a useful, minimally invasive technique to support the pre-mortem diagnosis of CNS disorders in dogs.

## Abbreviations

AIP: acute idiopathic polyradiculoneuritis
ANOVA: analysis of variance
cDNA: complementary DNA
CNS: central nervous system
CSF: cerebrospinal fluid
Ct: cycle threshold
GLM: general linear model
miRNA or miR: microRNAs
MRI: magnetic resonance imaging
MUO: meningoencephalitis of unknown origin
qRT-PCR: real-time quantitative PCR
RBC: red blood cells
SRMA: steroid-responsive meningitis-arteritis
SOD1: superoxide dismutase 1
